# Comparison of Pectin Layers for Nicotine Transdermal Patch Preparation

**DOI:** 10.15171/apb.2018.047

**Published:** 2018-08-29

**Authors:** Jirapornchai Suksaeree, Jessada Prasomkij, Kamon Panrat, Wiwat Pichayakorn

**Affiliations:** ^1^Department of Pharmaceutical Chemistry, Faculty of Pharmacy, Rangsit University, Muang, Pathum Thani 12000, Thailand.; ^2^Pharmaceutical Laboratory Service Center, Faculty of Pharmaceutical Sciences, Prince of Songkla University, Hat-Yai, Songkhla 90112, Thailand.; ^3^Department of Pharmaceutical Technology, Faculty of Pharmaceutical Sciences, Prince of Songkla University, Hat-Yai, Songkhla 90112, Thailand.

**Keywords:** Isolated pectin, Nicotine transdermal patches, Durian fruit, Cissampelos pareira, Krueo Ma Noy

## Abstract

***Purpose:*** The objective of the present investigation was to prepare and evaluate transdermal patches for nicotine.

***Methods:*** Pectin isolated from the hulls of Monthong durian or leaves of Krueo Ma Noy was used as a matrix membrane for the controlled release of nicotine and compared with commercial pectin. The mechanical properties, moisture uptake, and Fourier transform infrared spectra were characterized. The in vitro stability of these patches was evaluated and compared to commercial nicotine patches.

***Results:*** The mechanical properties of the patches made from isolated pectin were greater than those prepared from commercial pectin; brittle commercial patches were obtained after nicotine loading. The moisture uptake of the patches made with isolated pectin was in the range of 30.20-44.29%. There was no incompatibility between the ingredients of the nicotine transdermal patches or any degradation of the drug. The matrix layer made from isolated pectin controlled the nicotine release more effectively than did commercial nicotine patches. In addition, these patches were stable at in a refrigerator (approximately 4±2 °C) and at ambient temperature (approximately 30±2 °C) for 3 months, retaining 90% of the loaded nicotine.

***Conclusion:*** Our study suggests that using isolated pectin as the matrix layer should control the release of nicotine from transdermal patches.

## Introduction

Pectin, a natural biopolymer, is a structural heteropolysaccharide with a high molecular weight. It is found in the primary cellular walls and middle lamella in plant tissues such as the peels of citrus fruits or apples. It is used in pharmaceutical and food applications as a gelling agent, thickening agent, emulsifying agent, and stabilizer and as a source of dietary fibre that is free of additives. The gel formation of pectin is extensive with a low methoxy component. Its gel properties depend on various factors such as the concentration, pH, molecular size, and characteristics of the raw material, which in turn are determined by the source and the extraction conditions used. The chemical structure of pectin consists of linear α-(1-4)-D-galacturonic acid molecules.^1^ Commercial pectin is mostly extracted by treating the peels of apples or citrus fruits with hot dilute mineral acids. The appearance of commercial pectin is a white to light brown powder. Recently, several studies have reported a novel pectin isolated from the rinds and hulls of durian,^2-6^ peels of papaya,^7^ endocarps of *Citrus depressa*,^8^ peels of mango,^9^ and leaves of *Cissampelos pareira*.^10,11^

Durian or *Durio zibethinus* is known as the king of fruit in Thailand.‏ The durian‏ fruit is egg-shaped and large with a thick, spiky hull with an average weight of 1‏.5 ‏– 5‏.0 kg, depending on its source‏. Durian is readily found in Southeast Asia, especially Thailand, between May and August. The hulls of durian are disposed of as waste, which could lead to environmental problems.^3^ There has been recent interest in the hulls of durian as a valuable material of commercial importance instead of agricultural waste. In addition, both the water‏-insoluble and water‏-soluble pectin from the hull of durian are potential excipients for pharmaceutical applications.^2,3,5,6^

Krueo Ma Noy, Monoi, or *Cissampelos pareira* is a woody vine with leaves up to 30 cm in length. It is widely found in the north and the northeast of Thailand. It is used by indigenous people as a medicinal herb to treat a variety of ailments such as traumatic pain, asthma and dysentery.^12^ The root extracts of Krueo Ma Noy exhibit antitumor, antileukaemia, diuretic, and muscle relaxant properties.^13-15^ Singthong and co-workers recently studied the extraction method, structural characterization, and gelling properties of pectin from leaves of Krueo Ma Noy.^10,11^ Gelling occurred in aqueous solutions at 0.5 and 1.0% w/v at 5°C and room temperature, respectively.

Nicotine is a pyridine alkaloid derived from the tobacco plant.^16^ It is easily absorbed and permeates through the skin when applied topically.^17-19^ Nicotine patches are applied directly to the skin as an aide for cigarette smoking cessation. The patch is applied to dry, clean, hairless skin on the upper arm, upper chest, or hip; irritated, oily, scarred, or broken skin should be avoided. The various advantages of nicotine transdermal patches are to avoid first-pass metabolism, sustain a constant plasma concentration, increase drug bioavailability and efficacy, provide good patient compliance, and enable faster drug delivery termination by removing the patch compared to the oral drug administration route.^20-23^ Currently, nicotine transdermal patches are developed in many dosage forms such as film-forming solution,^24,25^ reservoir-type transdermal patches,^26^ and matrix-type transdermal patches.^27,28^

Thus, pectins isolated from both the hulls of durian and leaves of Krueo Ma Noy are of interest as a film-forming agent for a transdermal system for the delivery of nicotine, which has not yet been reported‏. However, in a recent study, we prepared nicotine transdermal patches using 5% w/w isolated pectin from the hulls of Monthong durian blended with low-protein natural rubber latex. It was found that this polymer blend can potentially control drug release from the patch.^29^ However, the effect of the different pectin types on the patch preparation was not studied; therefore, in this study, the different pectin types, including isolated pectin from the hulls of Monthong durian, isolated pectin from the leaves of Krueo Ma Noy, and commercial pectin, were compared at 2.5% w/w, and the stability of these patches was studied. The aim of this research was to prepare nicotine transdermal patches using 2.5% w/w isolated pectin from the hulls of Monthong durian, isolated pectin from the leaves of Krueo Ma Noy, and commercial pectin as a matrix layer with glycerine as a plasticizer. The mechanical properties, moisture uptake, and Fourier transform infrared (FTIR) spectra of the prepared nicotine transdermal patches were characterized. *In vitro* and stability tests were also performed to compare the isolated pectin to the commercial nicotine transdermal patch.

## Materials and Methods

### Materials

(-)-Nicotine (≥99%) was purchased from Merck (Germany). The hulls of Monthong durian and leaves of Krueo Ma Noy were collected from Chanthaburi and Surin Provinces, Thailand, respectively. Ethyl cellulose and diethyl phthalate were purchased from Sigma-Aldrich, USA. Commercial pectin from the peel of citrus fruits was purchased from VR Bioscience Co., Ltd, Thailand. Glycerine was purchased from Sigma-Aldrich, USA.

### Preparation and isolation of pectin

**Figure 1:**
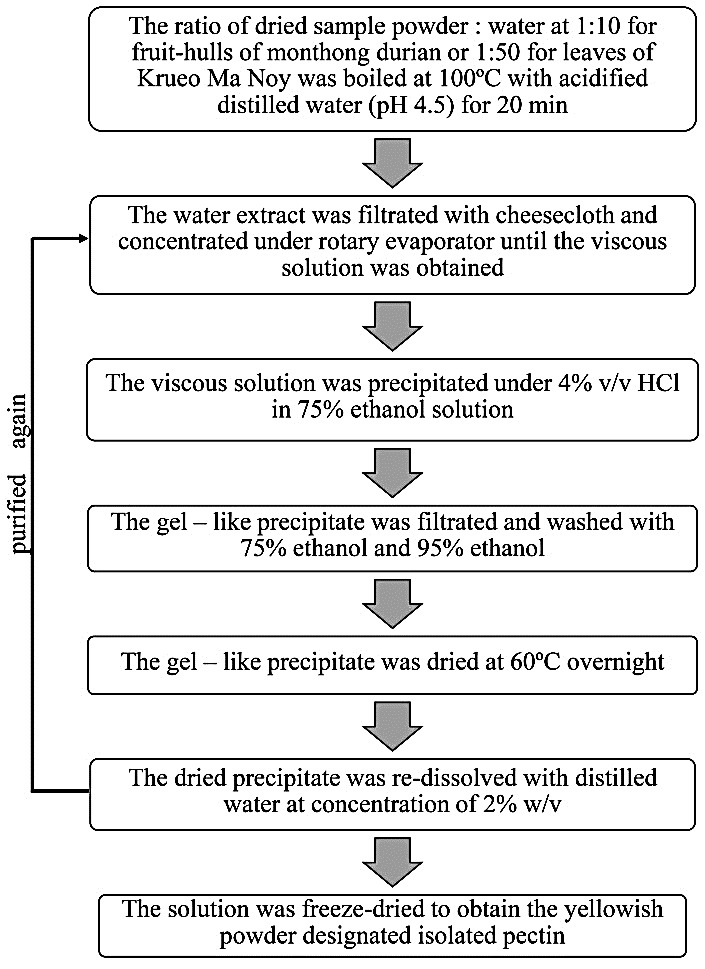
The process of isolating pectin from ‏the hulls of Monthong durian or leaves of Krueo Ma Noy

The isolated pectin was prepared from the hulls of Monthong durian or leaves of Krueo Ma Noy. The fruit-hulls and leaves were cleaned and dried in a hot-air oven at 60°C overnight and subsequently powdered. Then, the dried sample powder of the hulls or leaves was isolated as depicted in Figure 1.

### Preparation of nicotine transdermal patches

The backing layer was prepared by dissolving 10% w/w ethyl cellulose in acetone, and 20% w/w (based on dry weight of polymer) diethyl phthalate was used as a plasticizer. The plasticized ethyl cellulose solution was poured in to a Petri dish with an effective area of 70.88 cm^2^, and the solvent was evaporated. The 2.5% w/w isolated pectin was dissolved in distilled water, and 30% w/w glycerine (based on dry pectin powder) was slowly added as a plasticizer. Nicotine solution was dissolved in distilled water and dropped into the solution. Nicotine content initially loaded in the transdermal patches was 3.00 mg/cm^2^. A clear solution was formed. Fifteen grams of the mixtures was poured into a Petri dish containing a backing layer and dried by a hot-air oven at 70 ± 2°C for 5 hrs. Subsequently, the dry nicotine transdermal patches were peeled from the Petri dish and kept in a desiccator.

### Characterization of nicotine transdermal patches

#### Mechanical properties

The mechanical properties of the nicotine transdermal patches were tested using the TA.XT Plus Texture Analyzer (Texture Technologies Corporation and Stable Micro Systems, Ltd., USA) with a 500-g loaded cell. The film sample was cut into a 10 mm ´ 60 mm rectangular shape. The gauge length of the tested area was 10 mm, which was controlled at 10 mm/min of cross-head speed. Six samples of blank patches and nicotine transdermal patches were tested.

#### Moisture uptake study

The sample was cut into a 10 mm × 10 mm square and kept in a desiccator that equilibrated with saturated sodium chloride (75% relative humidity environment). The percentage of moisture was calculated by the following equation (1).

(1) $$\text{\%Moisture uptake = }\;\frac{{\text{(W}}_{\text{u}}\text{ -}\;{\text{W}}_{\text{0}}\text{)}}{{\text{W}}_{\text{0}}}\text{ ×100}$$

Where W_0_ is the initial weight of the sample and W_u_ is the constant weight of the sample.

#### FTIR study

The functional groups of ingredients were observed by the FTIR spectrometer (model: Nicolet 6700, DLaTGS Tetector, Thermo Scientific, USA). The scanning resolution was 4 cm^-1^ with 16 scans over a wavenumber region of 400 - 4000 cm^-1^.

#### The determination of nicotine content 

The sample was accurately weighed and extracted in 5 mL of distilled water by sonication for 30 minutes. The solution was filtered through the membrane. Then, 0.5 mL of solution was collected and diluted to 10 mL with distilled water. The nicotine content in each patch was determined with an HPLC by comparison with the validated calibration curve. The nicotine entrapment efficiency and loading capacity were calculated. Triplicate observations of each sample were measured.

#### In vitro study of nicotine from transdermal patches

The *in vitro* release of nicotine from the isolated pectin patches was measured with an adapted Franz-type diffusion cell (Hanson^®^57-6M, Hanson Research Corporation, USA) compared to a commercial nicotine patch (Nicotinell TTS-20; 1.75 mg/cm^2^). The effective diffusion area for study was 1.77 cm^2^. The patch was directly applied to the cellulose membrane (MWCO: 12,000-14,000, CelluSep^®^ T4, Membrane Filtration Product, Inc., USA) and used as a barrier between the donor and receptor compartments. The receptor compartment was filled with 12 mL isotonic phosphate buffer solution (PBS) with a pH of 7.4, the solution was stirred constantly at 100 rpm by magnetic stirrer, and the temperature was maintained at 37 ± 0.5 °C. One millilitre of receptor solution was withdrawn at 0.5, 1, 2, 3, 4, 6, 8, 12 and 24 hrs, and an equal volume of freshly prepared PBS was then replaced. The nicotine concentration in these samples was determined by the HPLC method. Triplicate observations of each sample were measured.

The* in vitro* skin permeation employed new-born pig skins as a barrier between the donor and receptor compartments. The new-born pig skins were purchased from a local pig farm in Chachoengsao Province. Hair, subcutaneous fat, and other extraneous tissues of the skins were trimmed with a scalpel. Then, the skins were washed and examined for integrity. They were stored at 4°C overnight and then soaked overnight in PBS before the permeation experiments. The *in vitro* skin permeation of nicotine from the blended patches was measured with an adapted Franz-type diffusion cell with an effective diffusion area of 1.77 cm^2^. The patch was directly applied on the epidermal side of the skin. The receptor compartment was filled with 12 mL of PBS at pH 7.4 and contacted the dermis side of the skin, the solution was stirred constantly at 600 rpm by a magnetic stirrer, and a temperature of 37 ± 0.5 °C was maintained. One millilitre of receptor solution was withdrawn at 0.5, 1, 2, 3, 4, 6, 8, 12 and 24 hrs, and an equal volume of freshly prepared PBS was then replaced. The nicotine concentration in these samples was determined by the HPLC method. Triplicate observations of each sample were measured.

The mechanism of *in vitro* release and skin permeation was calculated and fitted with different mathematical models given by equations (2-4).^30,31^ The classical squared correlation coefficient (r^2^) was calculated from the slope of each linear portion plot.

(2) $$Q_{t}=Q_{0}+K_{0}t$$

(3) $${\text{log Q}}_{\text{t}}{\text{=log Q}}_{\text{0}}{\text{+K}}_{\text{1}}\text{t}$$

(4) $$\frac{Q_{t}}{Q_{0}}=K_{H}\sqrt{t}$$

Where K_0_ is the zero constant rate (mg/h)

K_1_ is the first constant rate (mg/h)

K_H_ is the Higuchi’s constant rate (mg/√h

Q_t_ is the amount of nicotine released (mg) in time t (h)

Q_0_ is the initial amount of nicotine (mg) in the matrix-type transdermal patches

The HPLC analysis was performed with an Agilent 1260 Infinity system (Agilent Technologies, USA) using the UV detector at 260 nm. The reverse-phase ACE Generix5 C18 (4.6 mm × 150 mm, 5 µm particle size, DV12-7219, USA.) was used to separate nicotine. The mobile phase with a flow rate of 0.7 mL/min was 0.05 M sodium acetate:methanol (9:1 v/v) containing 1.3% triethanolamine, and the pH was adjusted to 4.2 with acetic acid. The sample injection was 10 µL. The calibration curve had a linearity of more than 0.9992 in 2-50 µg/mL of standard nicotine. The limit of detection and limit of quantitation were 0.21 and 0.75 µg/mL, respectively, of standard nicotine. The accuracy ranged from 95.56-104.85%, and the precision was less than 1.50%RSD.

#### Stability study of nicotine in transdermal patches

The nicotine patches were wrapped in aluminium foil and kept in the refrigerator (approximately 4±2 °C) and ambient (approximately 30±2 °C) temperatures for 3 months. At appropriate time intervals, the nicotine patches were collected to extract nicotine with distilled water. The remaining nicotine content was analysed using an HPLC.

## Results and Discussion

### Characterization of nicotine transdermal patches

#### Mechanical properties

**Figure 2:**
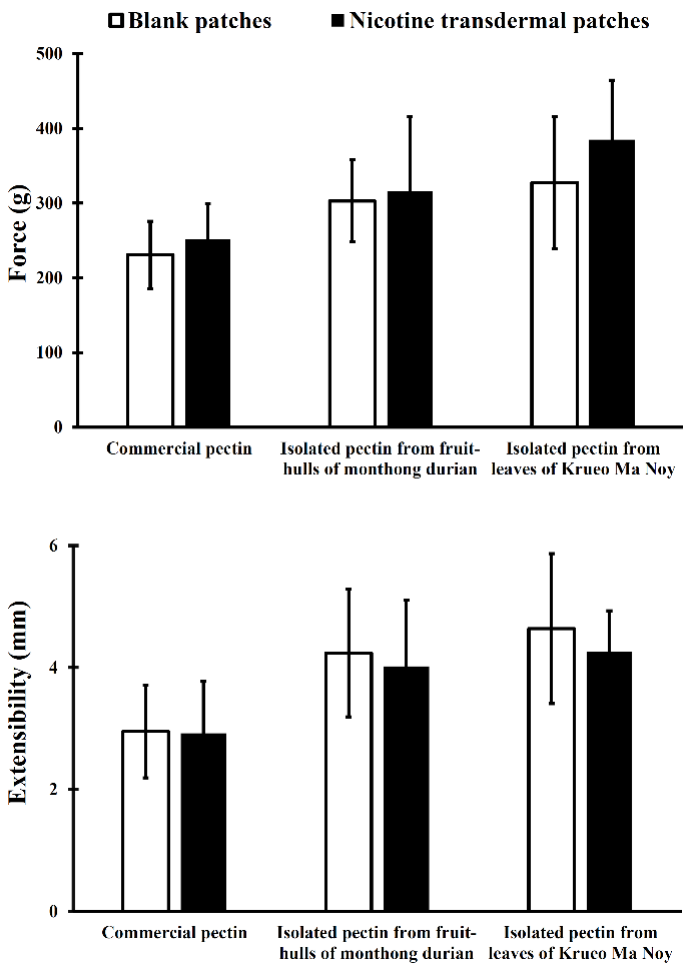
Mechanical properties of nicotine transdermal patches made from commercial pectin and isolated pectin (n = 6)

The thickness of the blank patches and nicotine transdermal patches was 102-125 µm as determined from five different positions on the patches. Their mechanical properties, tensile strength and elongation to break were investigated (Figure 2). The average tensile strength and elongation to break for the patches prepared with isolated pectin were greater than those prepared from commercial pectin. The addition of nicotine in the patches decreased these values, indicating that these patches were less extensible than the corresponding controls. The blank patches were easily removed from the Petri dish where they were cast; in contrast, the nicotine transdermal patches appeared stiff and glassy and were extremely adherent to the Petri dish. Thus, the addition of nicotine gives rise to brittle patches. Subsequently, the backing layer was used in studies to easily remove the patches from a Petri dish without observable damage.

#### Moisture uptake study

**Figure 3:**
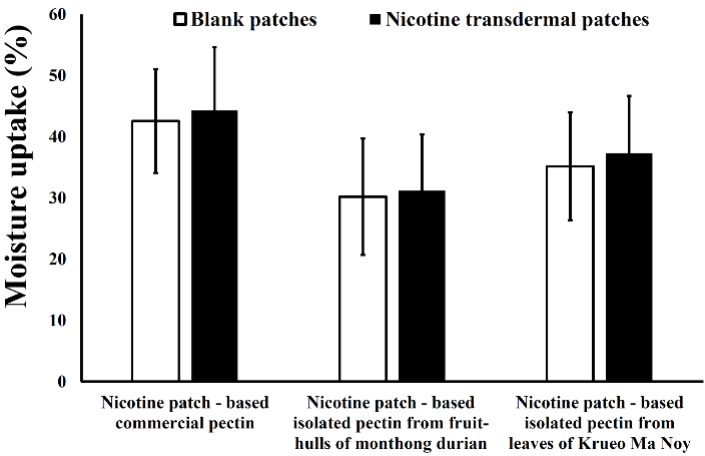
Moisture uptake of nicotine transdermal patches made from commercial pectin and isolated pectin (n = 6)

The moisture content of the transdermal patches made from isolated pectin was significantly lower than that of transdermal patches made from commercial pectin. The moisture uptake range for blank transdermal patches was 30.20-42.54%, and the moisture uptake range for nicotine transdermal patches was 31.19-44.29%. Pectin easily absorbs the moisture from the environment because it is hydrophilic; however, this behaviour is decreased when blended with a hydrophobic polymer.^29^ In addition, nicotine is a hygroscopic substance; thus, it also slightly increased the moisture uptake when compared to the blank transdermal patches (Figure 3). This result is related to the previous preparation of the nicotine transdermal patches, showing little increase in the moisture uptake after nicotine is loaded in the patches.^27,32^

#### FTIR study

**Figure 4:**
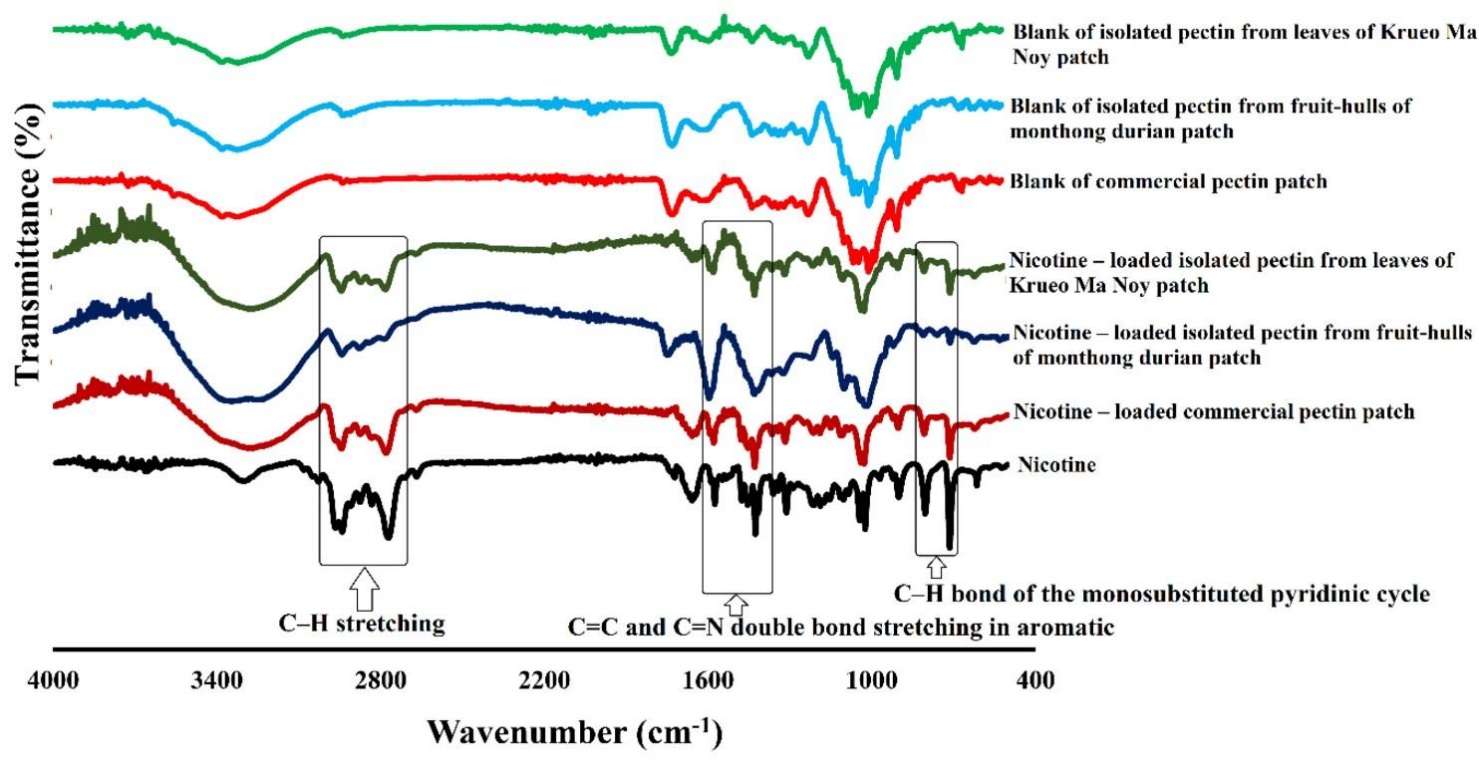
FTIR spectra of nicotine transdermal patches made from commercial pectin and isolated pectin

The FTIR analysis in the wave number range from 400 – 4000 cm^-1^ was used to evaluate the main functional groups of the drug and the polymer shown in Figure 4. Table 1 shows the main chemical groups for nicotine and blank patches given in Figure 4. The major chemical groups of polysaccharides were found in both commercial and isolated pectin, confirming that the polysaccharide extracted from the hulls of Monthong durian and leaves of Krueo Ma Noy was pectin. The main functional groups of these pectins are similar to those in previous publications.^33,34^ In the nicotine matrix patches, the main chemical groups of nicotine were 2900-2600, 1650-1575, and 900-714 cm^-1^, which represented C‏–H stretching, aromatic C=C and C=N double bond stretching, and C-H bond of the monosubstituted pyridinic cycle, respectively. There were no marked spectrum changes observed between the raw materials. Thus, the nicotine transdermal patches exhibited no incompatibility among any ingredients in the patches or degradation of the drug.

**Table 1:** The identification of FTIR spectra

**-**	**Wave number (cm^-1^)**	**Functional groups**
Nicotine	3303	The large peak of water ( it deals with a liquid )
	2966-2668	The C‏–H stretching
	1658	The aromatic C‏=C double bond stretching
	1575	The aromatic C‏=N double bond stretching
	901 and 714	The out-of-plane bending of the C‏–H bond of the monosubstituted pyridinic cycle
Blank of commercial pectin patch	3381	The O‏–H stretching
	‏2937	The C‏–H stretching
	1733	The C‏=O‏ stretching
	1618	The COO‏ asymmetric‏ stretching
	1438	The COO‏ symmetric‏ stretching
Blank of isolated pectin from fruit‏-hulls of Monthong durian patch	3326	The O‏–H stretching
	2940	The C‏–H stretching
	1731	The C‏=O‏ stretching
	1625	The COO‏ asymmetric‏ stretching
	1438	The COO‏ symmetric‏ stretching
Blank of isolated pectin from‏ leaves of Krueo Ma Noy patch	3320	The O‏–H stretching
	2936	The C‏–H stretching
	1735	The C‏=O‏ stretching
	1598	The COO‏ asymmetric‏ stretching
	1436	The COO‏ symmetric‏ stretching

### The determination of nicotine content 

The nicotine transdermal patches were extracted in distilled water and sonicated for 30 min. The nicotine content was 1.90±0.05, 1.79±0.06, and 1.84±0.06 mg/cm^2^ in the commercial pectin patch, isolated pectin from the hulls of Monthong durian patch, and isolated pectin from leaves of Krueo Ma Noy patch, respectively. The entrapment efficiencies of the nicotine-loaded commercial pectin patch, isolated pectin from hulls of Monthong durian patch, and isolated pectin from leaves of Krueo Ma Noy patch were 63.56±1.68%, 59.78±2.01%, 61.44±1.90%, respectively. The low nicotine content in the transdermal patches is probably due to the nicotine volatilizing in the drying process that forms the transdermal patches.

### In vitro study of nicotine from transdermal patches

**Figure 5:**
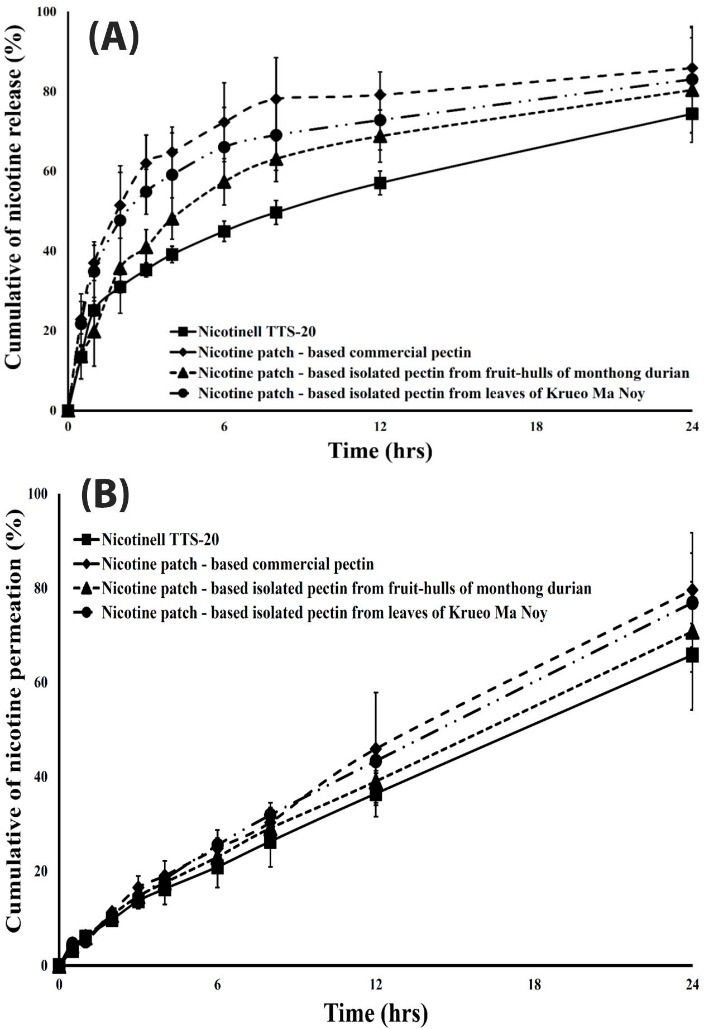
*In vitro* release (A) and skin permeation (B) of nicotine from transdermal patches made from commercial pectin and isolated pectin (n=3)

Figure 5A shows the *in vitro* release of nicotine from transdermal patches. The nicotine release was high during 0 – 10 hrs and reached steady-state after 10 hrs. The higher percentage of moisture uptake of the nicotine transdermal patches likely also provided more drug release. These results could be explained by the increased hydrophilicity of the nicotine transdermal patches. However, these nicotine release profiles from pectin patches were much higher than those of Nicotinell TTS-20 (commercial nicotine patch). The nicotine release significantly increased since nicotine could freely dissolve in the patches. The kinetics of the nicotine release from Nicotinell TTS-20 were similar to those of Higuchi’s model that accounted for the diffusion and produced a depleted release from the surface of the patch. Thus, the dissolution and erosion of the patch were not the only parameters that influenced the nicotine release from Nicotinell TTS-20. However, the dissolution and erosion mechanism affected the nicotine release from the patches made from pectin, which was confirmed by a first-order kinetics model (Table 2).

Although the release profiles of nicotine from the pectin patches were much higher than those of the Nicotinell TTS-20, the permeation of nicotine from these pectin patches (Figure 5B) did not differ from those of the Nicotinell TTS-20 due to the skin barrier of the stratum corneum.^35^ In addition, the pectin patches contained glycerine as a plasticizer, which enhanced the nicotine absorption into the skin. These results were similar to other results demonstrating the major effect on drug release and permeation.^27,32,36^ The permeation of nicotine into the skin from all pectin matrix patches exhibited zero-order kinetics, which was similar to that of the Nicotinell TTS-20 (Table 2). The *in vitro* release and skin permeation of nicotine from the patch depended on the type of matrix layer. Nicotine can be easily released and permeate from the matrix layer that is made from hydrophilic polymer more so than the matrix layer that is made from hydrophobic polymer. Because the hydrophilic polymer can absorb the moisture from the environmental and swell, it creates porosity in the patch channels to increase the nicotine release from the patch.^27,32,37^‏ In addition, the increase in the concentration of pectin layer that is reported by our previous publication^29^ was associated with an increase in *in vitro* release and skin permeation of nicotine from the patch compared to the findings in this paper. This difference is because the high-concentration pectin layer has greater swelling behaviour in the patch than the low-concentration pectin layer.

In conclusion, when the patches had a high moisture uptake, they exhibited high dissolution and erosion of hydrophilic substances and drug molecules from the matrix. In addition, nicotine is highly hygroscopic and dissolved by water penetrating through the patches; thus, the increased porosity of the channels led to greater release of the drug.^38,39^ Therefore, the water uptake behaviour of the matrix patches plays an important role during the early stages of drug release, providing a higher release rate and skin permeation rate.

#### Stability study of nicotine in transdermal patches

**Figure 6:**
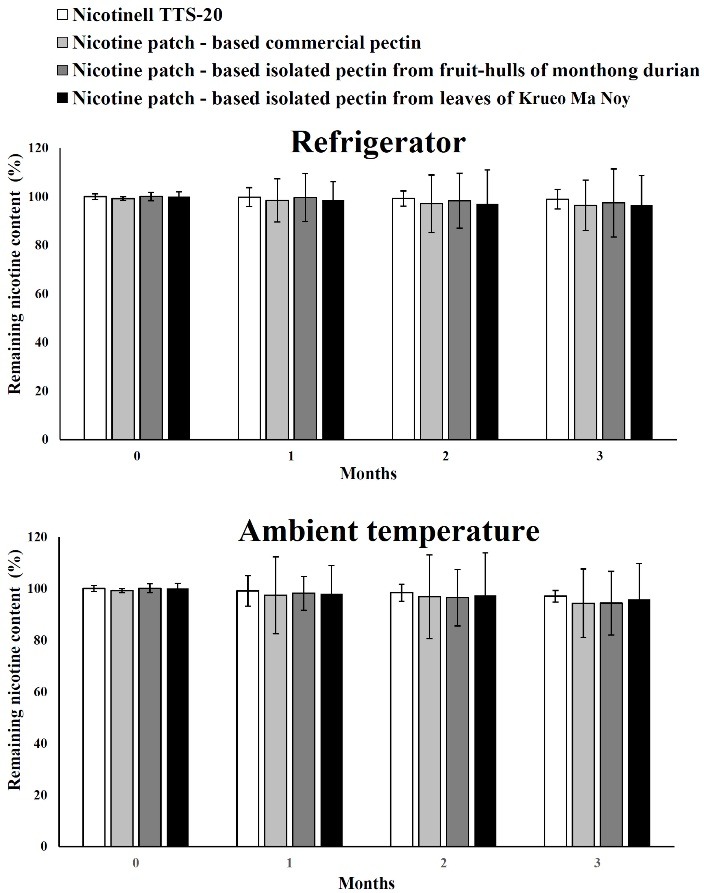
The remaining nicotine content in transdermal patches made from commercial pectin and isolated pectin after storage at refrigerator and ambient temperatures for 3 months (n=3)

The nicotine transdermal patches were evaluated for stability at 4 °C and ambient temperature. Because the nicotine quickly volatilized when kept at higher temperatures, 4 °C and ambient temperature were selected for this study. The shelf-life of nicotine content in transdermal patches remained greater than 90% (Figure 6). The final appearance of all nicotine transdermal patches was slightly brown compared with the initial preparation under visual observation.

**Table 2:** *In vitro* release and skin permeation kinetics of nicotine from transdermal patches

**-**	**Nicotine transdermal patches**	**Kinetic models**	**Equation**	**R** ^ 2 ^
*In vitro* release	Nicotinell TTS‏-20	Zero order	y =2.5913x + 21.313‏	0.7865
		First order	y = -0.0221x + 0.150	0.9363
		Higuchi‏’s model	y= 14.714x + 6.8345	0.9688
	Nicotine ‏– loaded commercial pectin patch	Zero order	y =2.719x + 38.895	0.7184
		First order	y = -0.0977x + 0.0539	0.9131
		Higuchi‏’s model	y =17.368x + 19.75	0.8080
	Nicotine ‏– loaded isolated pectin from fruit‏-hulls of Monthong durian patch	Zero order	y =2.9495x + 24.93	0.7013
		First order	y = -0.0221x + 0.1598	0.9363
		Higuchi‏’s model	y = 17.369x + 7.1765	0.9292
	Nicotine ‏– loaded isolated pectin from leaves of Krueo Ma Noy patch	Zero order	y = 2.6373x + 34.954	0.7742
		First order	y = -0.0221x + 0.1718	0.9363
		Higuchi‏’s model	y = 16.407x + 17.285	0.8490
*In vitro* skin permeation	Nicotinell TTS‏-20	Zero order	y = 2.6557x + 3.7694	0.9906
		First order	y = -0.0484x + 0.2269	0.9886
		Higuchi‏’s model	y = 13.301x - 7.4231	0.9493
	Nicotine ‏– loaded commercial pectin patch	Zero order	y = 3.2526x + 4.0204	0.9902
		First order	y = -0.0484x + 0.2626	0.9886
		Higuchi‏’s model	y = 16.265x - 9.6361	0.9460
	Nicotine ‏– loaded isolated pectin from fruit‏-hulls of Monthong durian patch	Zero order	y = 2.8523x + 4.2534	0.9900
		First order	y = -0.0484x + 0.2367	0.9886
		Higuchi‏’s model	y = 50.941 x - 2.0427	‏0.9512
	Nicotine ‏– loaded isolated pectin from leaves of Krueo Ma Noy patch	Zero order	y = 3.1435x + 4.0732	0.9899
		First order	y = -0.0484x + 0.2487	0.9886
		Higuchi‏’s model	y = 15.762x - 9.2112	0.9507

## Conclusion

The transdermal patches made from commercial pectin, isolated pectin from the hulls of Monthong durian, and isolated pectin from the leaves of Krueo Ma Noy had good film-forming properties. The mechanical properties of the patches made from isolated pectin were greater than those prepared from commercial pectin and had moisture uptake in the range of 30.20 – 42.54%. After nicotine loading, the patches became brittle, and the moisture uptake increased. Moreover, neither drug-vehicle interaction nor drug degradation occurred. The matrix layer made from isolated pectin exhibited controlled nicotine release from the patches but at a greater rate than commercial nicotine patches. The rate of release of the drug from the patches followed a first-order equation in which the drug release rate depended on its concentration. However, the skin permeation of nicotine from all pectin matrix patches fitted a zero-order equation (constant rate), which is the ultimate goal for transdermal drug delivery. The nicotine transdermal patches were stable at refrigerator and ambient temperatures for three months. From this study, we conclude that the matrix patches prepared from isolated pectin from the hulls of Monthong durian or isolated pectin from leaves of Krueo Ma Noy loaded with nicotine can be evaluated for their properties to control the release of nicotine from transdermal patches.

## Acknowledgments

The authors would like to acknowledge the Faculty of Pharmacy and the Research Institute of Rangsit University for financial support (Grant No.3/2560). The authors would like to express their gratitude to Professor J. Edward Moreton, for his assistance in the English language in this paper‏.

## Ethical Issues

Not applicable.

## Conflict of Interest

The authors declare that they have no conflicts of interest.
